# Research Confirms Effectiveness of Oral Health Preventive Practices

**DOI:** 10.1093/geroni/igab046.968

**Published:** 2021-12-17

**Authors:** Michael Dodds

**Affiliations:** Mars Wrigley, Chicago, Illinois, United States

## Abstract

Lack of insurance or funds for dental services, lack of access to dental offices, fear of dentists, and avoidance of dental offices during COVID can lead to oral health problems in older adults. Brushing, flossing, and drinking fluoridated water can protect teeth when dentists are unavailable. Limiting intake frequency of carbohydrates and chewing sugarfree gum after eating add protection. A recent systematic review and meta-analysis confirmed the effectiveness of sugarfree gum in reducing caries, in children and adults who chewed sugarfree gum compared with those who did not chew. Chewing sugarfree gum significantly reduced caries increment, with a prevented fraction of 28 percent, roughly equivalent to the prevented fractions for fluoride toothpastes and supplements. A follow-up systematic review provides further evidence that chewing sugarfree gum reduces the numbers of Streptococcus mutans in the oral cavity. Finally, chewing sugarfree gum could alleviate symptoms of xerostomia and may reduce caries.

